# The Implementation of Data-Driven Assessment into Laparoscopic Skills Training: A Systematic Review

**DOI:** 10.1177/15533506251336824

**Published:** 2025-05-05

**Authors:** Sem F. Hardon, Tim Horeman, Sophie J. M. Reijers, Linda J. Schoonmade, Freek Daams, Donald L. van der Peet

**Affiliations:** 1 Department of Surgery, 1209 Amsterdam UMC – VU University Medical Center, Amsterdam, The Netherlands; 2 Department of BioMechanical Engineering, Delft University of Technology, Delft, The Netherlands; 3 University Library, 545812 Vrije Universiteit Amsterdam, Amsterdam, Netherlands

**Keywords:** laparoscopy, simulation training, technical skills, curriculum design, patient safety

## Abstract

**Background:**

Technological innovations have significantly enhanced the objective assessment of technical skills in minimally invasive surgery, offering substantial potential for proficiency-based training. However, the integration of these innovative tools into surgical education curricula remains limited. This study aims to evaluate the adoption and implementation of data-driven assessment tools within laparoscopic simulation training.

**Methods:**

A systematic search of PubMed and Embase was conducted following PRISMA guidelines, identifying studies that employed objective assessments of technical skills in surgical training curricula. Eligible studies utilized data-driven assessment methods as part of structured training programs for surgical residents. A descriptive analysis was performed on the included studies.

**Results:**

From 2814 identified articles, 718 were eligible for full-text screening, and 35 studies met the inclusion criteria. These studies described the implementation of 14 different data-driven tools in laparoscopic skills training. Most tools focused on assessing instrument handling, measuring parameters such as motion speed, path length, and accuracy. Only three studies evaluated tissue handling skills using metrics like knot quality, tissue handling forces, and anastomotic integrity.

**Conclusions:**

The adoption of data-driven tools in laparoscopic simulation training is progressing slowly and exhibits considerable variability. Most technologies emphasize instrument handling, while tools for assessing tissue manipulation and force application are limited. To improve training outcomes, a combination of motion- and force-based assessment tools should be considered, enabling a more comprehensive evaluation of technical skills in minimally invasive surgery.

## Introduction

Since the introduction of minimally invasive surgery (MIS), surgical trainers try to optimize outcomes and patient safety by improving technical competency.^1-5^ Although MIS has become the mainstay surgical approach, the complexity of this technique requires a new set of technical skills that is associated with a longer learning curve. This set of skills comprises of bimanual dexterity, hand-eye coordination, depth perception in a two-dimensional screen, dealing with the fulcrum effect, and reduced haptic feedback compared to open surgery.^6-9^

During the learning curve for laparoscopic surgery most errors occur in the early phase of mastering psychomotor skills.^10-13^ Particularly in this phase of skill acquisition it is imperative to learn in a safe environment.^5,13,14^ To overcome this part of the learning curve before operating on real patients, simulation training has been developed.^15-18^ In contrast to Halsted’s apprenticeship-tutor model of ‘see one, do one, teach one’, simulation training enables skill acquisition and passing the learning curve of technical skills before commencing MIS in the operating room (OR).^5,19-22^

Assessment tools to quantify training progression and to evaluate technical competency have been developed over the years.^3,15,20,23-25^ These methods vary from assessment forms, which are often labour intensive and susceptible of bias, to automated objective assessments based on measurements and metrics. The rapid increase in technical innovations for metric-based assessment has led to the accumulation of evidence in the current literature.^15,24,26,27^ However, it remains unclear to what extent these validated objective assessment tools are being used in clinical practice. More specific, how objective metric-based assessment is adopted into laparoscopic skills training, moreover, which technical skills are being assessed to determine competency.

This systematic review aimed to investigate the current state of adoption and integration of data-driven assessment tools for technical skills in laparoscopic skills training.

## Material and Methods

A systematic search of published literature was conducted to identify all evidence on data-driven assessment tools for technical skills in simulation training for laparoscopic surgery. This review was conducted and reported in adherence to the Preferred Reporting Items for Systematic Reviews and Meta-Analysis (PRISMA) guidelines and the AMSTAR-2 (A MeaSurement Tool to Assess systematic Reviews) checklist (*Supplemental files*).^28,29^

### Search Strategy

A comprehensive search was performed in the bibliographic databases PubMed and Embase from inception to 1 December 2023, in collaboration with a health science librarian (LS). Search terms included controlled terms (MeSH in PubMed and Emtree in Embase), as well as free text terms. The following terms were used (including synonyms and closely related words) as index terms or free-text words: ‘laparoscopy’ and ‘technical skills’ and ‘curriculum' and ‘evidence based’ (*Supplemental files*). The search was performed without date or language restrictions. Duplicate articles were excluded by the librarian (LS) using Endnote x19 (Clarivate^tm^).

### Study Eligibility

Studies were considered eligible for inclusion when reporting on four major categories: (i) laparoscopic training for surgical residents, (ii) training and assessment of technical skills, (iii) existing and implemented skills training courses or curricula, and (iv) validated objective measurements and metric generated by assessment tools. The studies had to include trainee data, described in randomized controlled trials (RCT), case-control studies, and prospective or retrospective cohort studies (NRSI), which had to be written in English or Dutch. Since this review focusses on the use of data-driven assessment in general, there was no preference towards RCT or NRSI.

#### Trainees

This review focused on technical skills, rather than cognitive and other skills (anatomical knowledge, knowledge of procedural order, and surgical decision-making) needed for laparoscopic surgery. Since basic laparoscopic skills are usually acquired first, studies were selected that describe surgical residents in the post-graduation year (PGY) 1 and 2. However, at the beginning of laparoscopy, simulation based training was reserved for more senior residents. Besides, because training goals and entrusted professional activities per PGY differ among countries and residency programs, also more senior residents could be included if they conducted basic laparoscopic skills training. The studies had to report on conventional laparoscopic surgery in one of the following surgical sub-specialisms: general, gastrointestinal, gynecology, urology, or pediatrics. All studies that described medical students, surgeons, or non-medical participants were excluded, since these by definition did not described a training curriculum for surgical residents.

#### Training

To evaluate the use of metrics for assessment of technical skills, studies that describe basic laparoscopic skills training were included. The training goals were acquisition and development of hand-eye coordination, dexterity, basic instrument handling and tissue manipulation. Therefore, studies had to utilize fundamental of laparoscopic skills (FLS)-like training tasks and laparoscopic suturing. To exclude more complex and procedure-like training task, only, only inanimate training modules, such as box trainers (non-VR) and virtual reality (VR) trainers were included. More advanced endoscopic techniques, such as robotic-assisted surgery, single-incision laparoscopy, natural orifices transluminal endoscopic surgery, flexible endoscopy, or arthroscopy, objective parameters hold different potentials to any of these techniques, concerning instrument movement and tissue handling skills. Therefore, these techniques were excluded. Studies reporting on veterinary surgery were also excluded.

#### Assessment

The described assessment tool had to be integrated into an existing skills course, training, or curriculum, and not only be used as part of an experiment or validation study. To be eligible for inclusion, studies had to report on data-driven assessment tools. These are automated sensor-based systems or devices that measured and recorded continuous variables that represented technical skills. This review describes technology-enhanced, data-driven assessment tools, using single or combined metric assessment. Studies that reported only time to complete a task (task efficiency) measured by stopwatch, counted errors, or assessment forms filled in by assessors, or a combination of these non-automated assessment tools, were excluded.

### Study Selection

After the removal of duplications, the remaining articles were considered for inclusion based on the title and abstract. These articles were analyzed independently and in duplicate by two different reviewers (SH and SR) according to the PRISMA standards, and using Covidence software (Veritas Health Innovation, Melbourne, Australia). Conflicts on inclusion or exclusion were resolved by consensus. After the title and abstract screening, a full-text screening was performed.

### Data Extraction

At first, demographic data about the author, the year of publication, and the journal were collected. The country of the corresponding author indicated where the described training was conducted. Following, data about the trainees and the skills training curricula were collected. This included surgical specialization and the experience level of the trainees expressed in the post-graduation year (PGY). Important aspects of the skills training included the type of training system (hands-on box training using real instruments vs virtual reality (VR) system), the specification of the system that was used, and the protocol content (training tasks and training duration). Then, the most relevant data for this review were gathered, comprising all information about data-driven assessment tools. This included the systems for objective and metric-based assessment, the metrics that were measured and recorded, and the specific technical skills that were assessed during the skills training. To evaluate and appraise the value of each assessment tool, we screened for (pre-existent) validity evidence studies, if the authors had referred to this, and, if the reference was provided.

## Results

**Figure 1.**
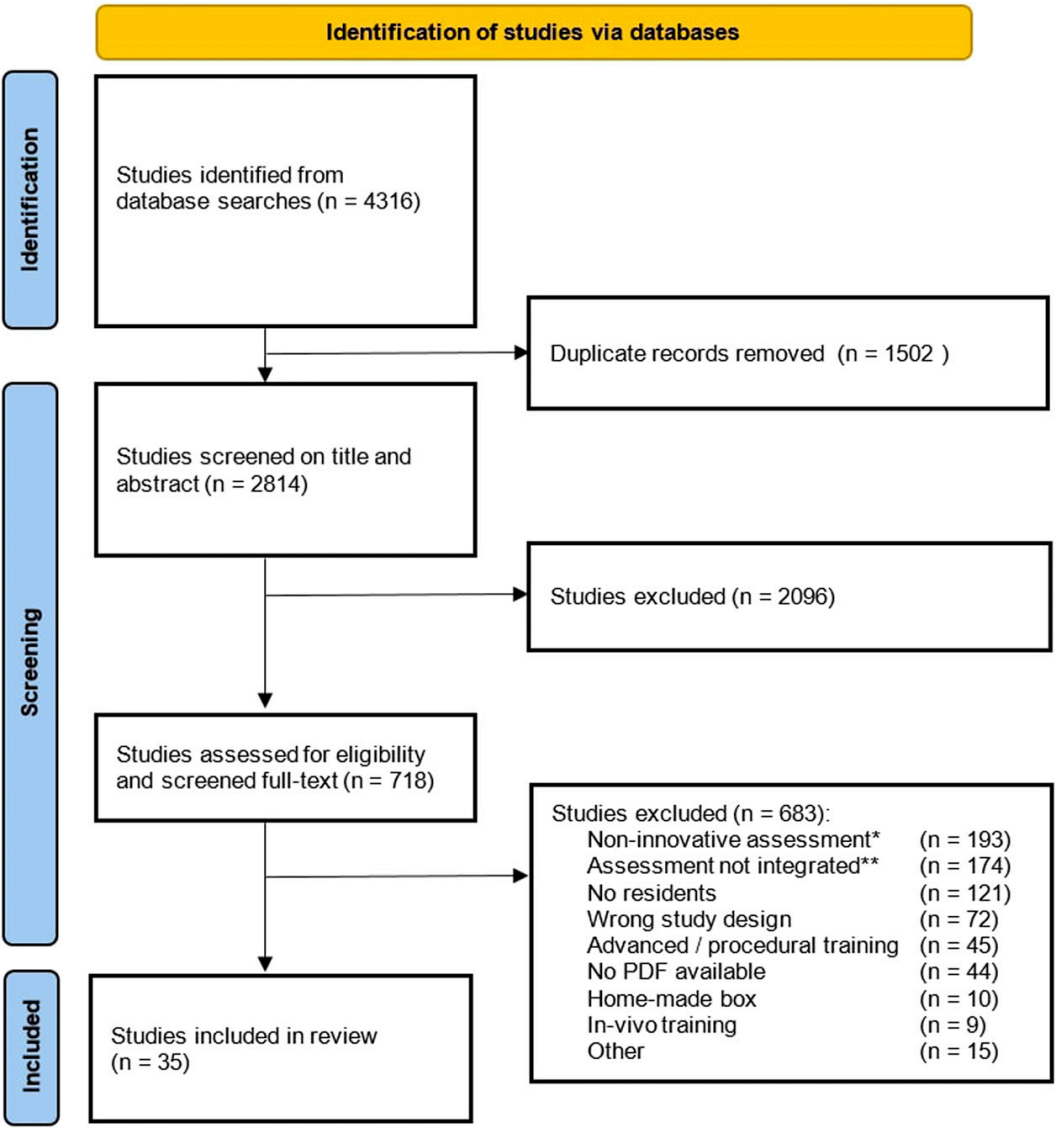
Study flowchart.

The search yielded a total of 4316 references. After removing duplicates, 2814 unique studies remained. Those which did not meet the inclusion criteria based on title and abstract screening (n = 2096) were excluded. The full- text of the remaining studies (n = 718) underwent qualitative analysis and were assessed for eligibility. Of these, we found that 193 studies (27%) described assessment that was not technology-enhanced and reported only time parameters, assessment form, or a combination. A total of 174 studies (24%) reported on data-driven assessment tools, but mainly in experiments and validation studies, and not adopted into a skills training curriculum for surgical residents. The third large group of 121 studies (17%) described data-driven assessment incorporated in a course, but not for surgical residents (ie, medical students, surgeons, non-medical participants). The full-text screening resulted in 35 studies (5%) that were included (Figure 1).

### Study Characteristics

**Figure 2.**
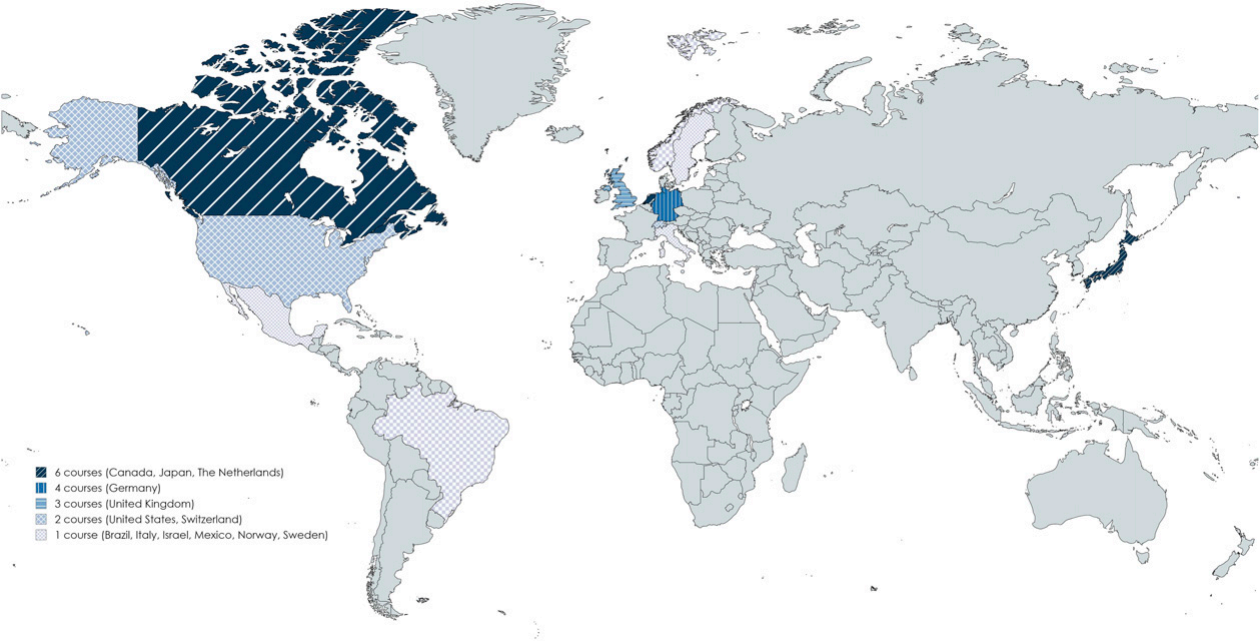
Areas of implementation of data-driven assessment tools.

**Figure 3.**
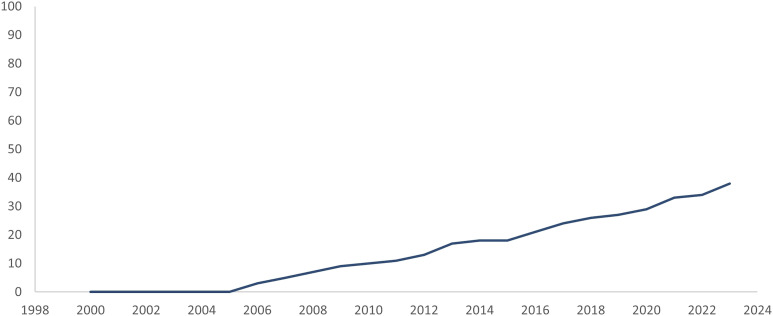
Adoption and integration of data-driven assessment tools into skills training curricula.

Between 2006 and 2023 laparoscopic skills training with integrated objective assessment tools was described in twelve countries (Figure 2), with a gradual increase over the past two decades (Figure 3).

#### Trainees

A total of 2618 surgical residents, median 27 (range 6 - 914), were trained and assessed by data-driven assessment tools for laparoscopic skills training (Table 1). Their surgical specialization was either general surgery (27 studies, 77%), urology (9 studies, 26%), gynecology (8 studies, 23%), pediatric surgery (2 studies, 6%), other (2 studies, 7%), or not specified (NS) (3 studies, 10%). Sixteen studies reported on trainees PGY 1 or 2, and nine studies reported also on more senior residents (up to PGY 6). In eleven studies the PGY was not specified.

**Table 1.** Laparoscopic Skills training Curricula.

Author	Year	Trainees			Training			
		N	Sub-Specialty^a^	PGY	Modality	Device	Tasks	Duration
Aggarwal et al.^52^	2006	23	General	1-2	Hands-on	Box trainer (NS)	Basic laparoscopic skills	2 days
Asencio et al.^30^	2018	41	General	NS	Hands-on	EVA II generation ETX A1 LAP (pro delphus)	Basic laparoscopic skills	2 days
Boettcher et al.^31^	2021	14	Pediatric surgery	1-4	Hands-on	Pulsating organ perfusion trainer (optimist)	Suturing	1 day
Cesanek et al.^32^	2008	20	General, gynecology	1-6	Hands-on	Szabo-Berci-Sackier (Karl Storz)	Basic laparoscopic skills, suturing	NS
Dubrowski et al.^53^	2007	24	General	1	Hands-on	USSC laptrainer (US surgical corp)	Suturing	NS
El-Beheiry et al.^54^	2017	49	General, gynecology, urology	1	VR	LAP mentor (simbionix)	Basic laparoscopic skills	2 days
van Empel et al.^55^	2013	72	General, gynecology, urology	2-6	Hands-on	Lapstar (camtronics)	Suturing	6 weeks
Feifer et al.^56^	2008	15	Urology	1-5	Hands-on	ProMIS (haptica)	Basic laparoscopic skills, suturing	2 days
Franco-González et al.^57^	2023	24	Pediatric surgery	1-4	Hands-on	EndoVis	Suturing	1 day
Giannotti et al.^58^	2013	42	General	1-2	VR	Wii (nintendo)	Basic laparoscopic skills	4 weeks
Grantcharov et al.^59^	2009	37	NS	1-2	VR	MIST-VR (mentice)	Basic laparoscopic skills	4 weeks
Hackethal et al.^60^	2006	61	NS	1-5	VR	MIST-VR (mentice)	Basic laparoscopic skills	4 days
Hagelsteen et al.^61^	2016	10	NS	1-5	Hands-on/VR	Box trainer (NS); simball box (G-coder systems), LapSim (surgical sciences)	Suturing	3 days
Handelman et al.^62^	2019	27	General, gynecology, urology	NS	Hands-on	Box trainer (NS)	Basic laparoscopic skills	NS
Hardon et al.^33^	2018	6	General, urology	1	Hands-on	Lapstar (camtronics)	Basic laparoscopic skills	3 weeks
Hardon et al.^12^	2021	24	General surgery, urology, other	1	Hands-on	Lapstar (camtronics)	Basic laparoscopic skills	3 weeks
Hassan et al.^63^	2006	44	General	NS	Hands-on	Box trainer (NS)	Basic laparoscopic skills , suturing, procedures	3 days
Johnson et al.^64^	2022	34	General, gynecology, urology, other	1-2	Hands-on	Box trainer (VTI medical)	Basic laparoscopic skills , suturing	6 weeks
Lehmann et al.^65^	2013	74	General	1-5	VR	Box trainer (NS) + other	Suturing, procedures	7 days
Mansoor et al.^66^	2020	22	General	NS	Hands-on	eoSim (eoSurgical)	Basic laparoscopic skills , suturing	NS
Masour et al.^67^	2012	48	General	NS	Hands-on/VR	Box trainer (NS)	Suturing, procedures	3 days
Paquette et al.^68^	2017	13	Gynecology	1-2	VR	LAP mentor II (simbionix)	Basic laparoscopic skills	8 weeks
Pastewski et al.^69^	2020	14	General	1-2	Hands-on	Box trainer (NS)	Basic laparoscopic skills	NS
Rahimi et al.^34^	2023	29	General, urology, other	1	Hands-on	Lapron (forcesense)	Basic laparoscopic skills	3 weeks
Rahimi et al.^35^	2023	42	General	1	Hands-on	Lapron (forcesense)	Basic laparoscopic skills	3 weeks
Rosenthal et al.^70^	2007	307	General	1-5	Hands-on/VR	Box trainer (NS)	Basic laparoscopic skills, procedure	NS
Sarker et al.^71^	2013	34	General	NS	Hands-on/VR	Szabo-Berci-Sackier (Karl Storz)	Basic laparoscopic skills , suturing, procedures	3 days
Takeda et al.^72^	2016	4	Gynecology	2	VR	LAP mentor (simbionix)	Basic laparoscopic skills	8 weeks
Takeoka et al.^36^	2017	12	General	3-12	Hands-on	Endowork pro II (kyoto kagaku) + other	Suturing	2 days
Tanoue et al.^73^	2010	194	General	NS	Hands-on/VR	Endowork Pro II (kyoto kagaku), procedicus MIST (mentice), LapSim (surgical science), LAP mentor (simbionix) + other	Basic laparoscopic skills , suturing	2 days
Tomikawa et al.^74^	2016	914	General, gynecology, urology	9±7	Hands-on/VR	Endowork Pro II (kyoto kagaku), procedicus MIST (mentice), LapSim (surgical science), LAP mentor (simbionix) + other	Basic laparoscopic skills , suturing	2 days
Uemura et al.^75^	2014	27	General	NS	Hands-on	Box trainer (NS)	Suturing	1 day
Verdaasdonk et al.^76^	2009	22	General	NS	VR	Simendo (deltatech)	Basic laparoscopic skills	12 weeks
von Websky et al.^77^	2012	286	General	1-2	VR	LAP mentor (simbionix)	Basic laparoscopic skills	NS
Yamaguchi et al.^78^	2011	9	General	NS	Hands-on	Box trainer (NS)	Suturing	1 day

^a^Other subspecialties included residents from plastic and reconstructive surgery, cardiothoracic surgery, orthopedic surgery, otolaryngology, and neurosurgery, that trained laparoscopic skills as part of their residency training program.

#### Training

Regarding the training modalities, 20 studies (57%) described the use of a hands-on box trainer (non-VR), 9 studies (26%) described VR trainers, and 6 studies (17%) investigated a combination of both modalities (Table 1). There were 19 different devices used for training, that were described in 25 studies. The most frequently used devices were the LAP mentor (/ LAP mentor II) (Simbionix), MIST (/ MIST- VR) (Mentice), Lapstar (Camtronics), Lapsim (Surgical Science), and the Endowork pro (Kyoto Kagaku). These devices were all used for VR training, except for the Lapstar (Camtronics) hands-on box trainer. Eleven studies did not specify the device that was used for training.

#### Assessment

**Figure 4.**
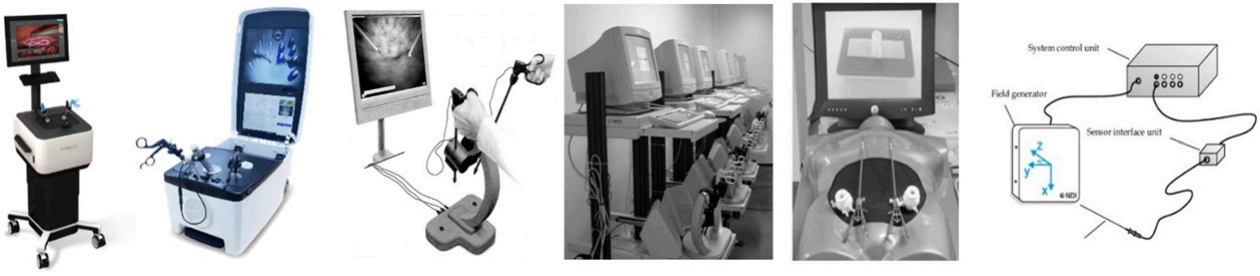
Most frequently used data-driven assessment tools (LAP mentor (Simbionix), Forcesense (Medishield/Forcesense), LAP sim (Surgical Science), MIST-VR (Mentice), ProMIS (Haptica), Aurora (Northern Digital)).

Similar to the training devices, there was also a large variation in the measurement systems used for objective parameter-based feedback. Four studies used a system that was not specified (NS). In the remaining 31 studies, 20 different systems for data-driven assessment were used and reported (Table 2). The most frequently used systems were LAP mentor (Simbionix), Forcesense (Medishield / Forcesense), LAP sim (Surgical Science), MIST VR (Mentice), ProMIS (Haptica), and Aurora (Northern Digital) (Figure 4).

**Table 2.** Assessment Tools and Validity Evidence.

Author	Assessment				Validity Evidence	
	System	Metric	Technical Skills	Additional^a^	Previous Validation^b^	Year
Aggarwal et al.^52^	Isotrak II (polhemus)	Path length (mm), movements (no.)	Instrument handling	Time, errors	Construct	2002
					Concurrent	2005
Asencio et al.^30^	Universal Testing Machine 5965 (Instron)	Maximum breaking strength (N)	Suture integrity	Time	Not reported	-
	Pachymeter (kingtools)	Knot loosening (mm)	Suture integrity	-	Not reported	-
Boettcher et al.^31^	Tensiometer (NS)	Maximum breaking strength (N)	Suture integrity	Time, knot quality, accuracy, performance	Content	1995, 2009
Cesanek et al.^32^	ProMIS (Haptica)	Path length (mm), smoothness (jerk cm/sec3), movements (no.)	Instrument handling	Time, errors	Construct	2005
	Sutures (U.S. Surgical corporation)	Knot loosening (mm)	Suture integrity	-	Not reported	-
Dubrowski et al.^53^	Isotrak II (polhemus)	Path length (mm), (speed mm/s), movements (no.)	Instrument handling	Time, errors	Construct	2002
					Content	2002
El-Beheiry et al.^54^	LAP mentor (simbionix)	Path length (m)	Instrument handling	Time, usage time	Not reported	-
van Empel et al.^55^	TrEndo (TU delft)	Path length (mm), depth perception (mm), angular area (deg^2^), volume (mm^3^)	Instrument handling	Time	Construct	2006, 2012
					Content	2012
					Face	2012
Feifer et al.^56^	ProMIS (haptica)	Path length (NS), smoothness (NS), movements (NS)	Instrument handling	Time	Construct	2005, 2006
Franco-González et al.^57^	EndoVis (NS) eZ430-Chronos module (Texas instruments)	Path length (m/s^2^), depth perception (m/s^2^), speed (mm/s), acceleration (mm/s^2^), smoothness (mm/s^3^), economy of area (ratio), economy of volume (ratio), angular length (°), response orientation (°), submovements (no.)	Instrument handling	Time	Construct	2015
	Triaxial accelerometer (bosch Sensortec)				Content	2013, 2015
					Face	2015
Giannotti et al.^58^	LAP mentor (simbionix)	Path length (NS), movements (no.)	Instrument handling	Time, errors	Not reported	
Grantcharov et al.^59^	MIST-VR (mentice)	Economy of motion score (NS)	Instrument handling	Time, errors	Construct	1998, 2003
					Content	1998
Hackethal et al.^60^	MIST-VR (mentice)	Economy of motion score (NS)	Instrument handling	Time, errors	Construct	1998, 1999, 2001 (2), 2002, 2003, 2004
					Content	1999, 2001 (2), 2002, 2004
Hagelsteen et al.^61^	Simball (surgical science)	Speed (cm/s), angular distance (radians), acceleration (mm/s^2^), smoothness (μm/s^3^)	Instrument handling	Time	Not reported	-
Handelman et al.^62^	Cut analysis laparoscopic simulator software (NS)	Smoothness (SD), dexterity (skewness, number of peaks, pixel area)	Instrument handling	-	Not reported	-
Hardon et al.^33^	ForceSense (Medishield)	Maximum force (N), mean force (N), force penalties (no.), path length (mm)	Tissue manipulation, instrument handling	Time, errors	Construct	2012, 2014
					Content	2014
					Concurrent	2014
Hardon et al.^12^	ForceSense (medishield)	Maximum force (N), maximum impulse (Ns), mean force (N), SD force (N), force volume (N^3^), Path length (mm)	Tissue manipulation, instrument handling	Time	Construct	2012, 2014, 2018
					Content	2014, 2018
					Concurrent	2014, 2018
					Face	2018
Hassan et al.^63^	LapSim (surgical science)	Path length (m) and angular path (°)	Instrument handling	Time, errors	Construct	2003, 2005
					Content	2002
Johnson et al.^64^	Electromagnetic sensors (ascension technology corporation)	Path length (mm), hand movements (no.)	Instrument handling	Time, errors	Construct	2015
					Content	2015
Lehmann et al.^65^	LapSim (surgical science)	Path length (m), instrument depth (mm)^d^, angular path (°)	Instrument handling	Time, errors	Construct	2007, 2008
					Content	2002, 2008
Mansoor et al.^66^	SurgTrac (eoSurgical)	Path length (m), distance between tips (cm), speed (mm/s), acceleration (mm/s^2^), smoothness (mm/s^3^)	Instrument handling	Time, errors	Construct^c^	2013, 2014, 2015
					Concurrent^c^	2013, 2015
					Content^c^	2013, 2015
Masour et al.^67^	Immersion VR (Immersion medical)	Path length (m)	Instrument handling	Time, errors	Not reported	-
Paquette et al.^68^	LAP mentor II (simbionix)	Economy of motion score (NS), instrument depth (NS)^d^	Instrument handling	Time, errors	Construct	2007, 2008
Pastewski et al.^69^	Motion-track software (NS)	Velocity (NS), acceleration (NS)	Instrument handling	Time, errors	Construct	2014, 2015
Rahimi et al.^34^	ForceSense (forcesense)	Maximum force (N), maximum impulse (Ns), mean force (N), SD force (N), force volume (N^3^), Path length (mm)	Tissue manipulation, instrument handling	Time, errors	Construct	2012, 2014, 2018, 2021
					Content	2014, 2018
					Concurrent	2014, 2018
					Face	2018, 2021
Rahimi et al.^35^	ForceSense (forcesense)	Maximum force (N), path length (mm)	Tissue manipulation, instrument handling	Time	Construct	2012, 2014, 2018, 2021
					Content	2014, 2018
					Concurrent	2014, 2018
					Face	2018, 2021
Rosenthal et al.^70^	LS 500 (xitact S.A.)	Path length (m)	Instrument handling	Time, errors	Construct	2003
Sarker et al.^71^	LAP mentor (simbionix)	Path length (cm), movements (no.)	Instrument handling	Time, errors	Construct	2006, 2007, 2009
Takeda et al.^72^	LAP mentor (simbionix)	Path length (cm)	Instrument handling	Time	Construct	2007
Takeoka et al.^36^	Hybrid simulator (NS) (kyoto kagaku co)	Maximum pressure resistance (kPa)	Anastomotic integrity	Time, errors	Construct	2015
Tanoue et al.^73^	LapSim (surgical science)	Path length (m), instrument depth (mm)^d^	Instrument handling	Time, errors	Construct	2005 (3), 2007
Tomikawa et al.^74^	3D electromagnetic system (aurora northern digital)	Path length (mm), speed (mm/s)	Instrument handling	Time, errors	Not reported	-
Uemura et al.^75^	Magnetic tracking sensor (NS)	Smoothness (center of gravity), stability (periodic orbit)	Instrument handling	-	Not reported	-
Verdaasdonk et al.^76^	SIMENDO (deltatech)	Path length (NS)	Instrument handling	Time, errors	Construct	2006, 2007
					Content	2006
					Face	2006
					Concurrent	2006
von Websky et al.^77^	LAP mentor (simbionix)	Path length (cm), speed (cm/s)	Instrument handling	Time, errors	Face	2007
					Criterion	2008
					Construct	2008, 2009
Yamaguchi et al.^78^	3D electromagnetic system (aurora northern digital)	Path length (mm), speed (mm/s)	Instrument handling	Time, errors	Not reported	-

^a^Errors: human observations, counted errors, task checklist or assessment forms, and non-automated video analysis.

^b^Only references provided by the authors were used and described.

^c^Validity evidence obtains in eoSim with InsTrac software.

^d^Tissue damage expressed as instrument handling parameter (tissue impression, depth perception).

Thirty-two (91%) of the studies reported on instrument handling skills, most often presented as path length, speed, or smoothness, with units varying between studies.

Eight studies (20%) reported on tissue handling assessment or tissue integrity, presented as

- suture integrity: breaking strength^30,31^ or knot loosening.^30,32^.
- tissue manipulation and tissue integrity: maximum forces, mean force, force penalties.^12,33-35^.
- anastomotic integrity: air pressure leakage.^36^

#### Validity evidence

**Figure 5.**
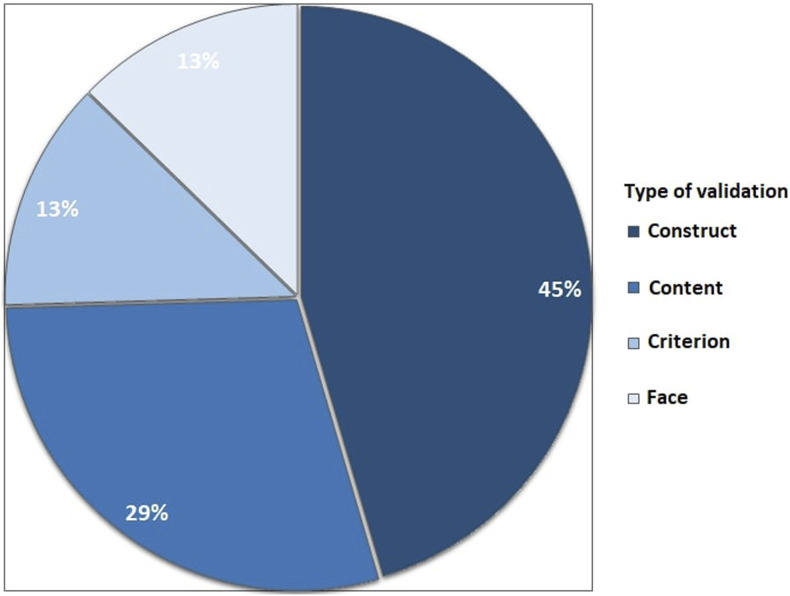
Reported validity evidence.

Eleven (31%) studies used a data-driven assessment tool that was not validated. The remaining 24 studies reported on assessment tools with previously established validity evidence, with a total of 55 references to the literature (Figure 5). These studies reported on construct validity (25 papers), content validity (16 papers), face validity (7 papers), and the remaining papers reported on a form of criterion validity (eg, concurrent validity) (7 papers).Some studies referred to multiple studies (from 2 up to 7 references) to prove validity evidence for construct, content, and criterion validity.

## Discussion

This systematic review shows that over 700 studies have described objective assessment tools for technical skills in laparoscopic skills training. However, most studies reported on skills laboratory experiments and validation studies, and data was often obtained by testing medical students. The vast majority of these objective assessment tools for surgical skills are not integrated into skills training. When assessment tools have been integrated into curricula, these are often conventional, hand-operated, and often unidimensional assessment tools, such as time to complete tasks, counted errors, or assessment forms.

Since the introduction of MIS, assessment forms and rating scales such as the Objective Structured Assessment of Technical Skills (OSATS) have been a reliable tool for the qualitative assessment of a procedural performance for over two decades, but are time-consuming for mostly, costly, senior assessors.^23,24,37,38^ Especially when considering that the assessment of skills should be part of a continuous curriculum with repetitive training.^12,15,21,33,34^ Bonrath et al concluded that, when used to identify intraoperative errors, assessment forms can be arbitrary and subjective. This resulted in the complexity of scale design and limited use outside the experimental setting.^25^ Using OSATS, surgeons can assess time, and instrument movements, and can estimate the damage to the tissue. Yet, no quantitative information on the actual effect that these instruments have on the tissue is provided.

With training shifting away from the patient into a simulation-based environment, trainers and researchers sought valid, reliable, and feasible alternatives. Rapid technological innovations led to the development of systems to identify errors and areas to improve technical skills, and numerous systems were validated that could quantify technical skills.^3,15,39^ The results show that, to date, 98% of the studies that used data-driven assessment tools in clinical practice, also used *time to complete a training task* as the main parameter for efficiency and technical competency. The majority of these studies also used counted errors or subjective hand-operated forms. Motion analysis parameters, such as instrument path length, speed, and smoothness, were most frequently described (93% of studies).

These parameters represent efficient and accurate instrument handling, rather than tissue manipulation skills. A recent study even found that when trainees focus only on the time to complete the tasks, the quality of surgical performance in terms of error rate and force applied to the tissue is impaired.^40^ Few studies reported new parameters of a different nature. In four studies, data-driven assessment tools were used to assess (i) suture integrity and knot quality based on the force to open the knots (in Newton) or knot loosening (in millimeters),^30,32^ (ii) tissue manipulation skills based on the force applied to the tissue (in Newton )33, and (iii) anastomotic integrity based on air leakage pressure (in Kilopascal).^36^

Validity evidence of force-based assessment is accumulating, and clinical relevance concerning patient safety has been shown.^8,12,41-44^ Studies found that excessive tissue handling and high grasping forces can lead to severe complications, such as tissue damage and even rupture, and threshold for safety between 1.3 Newton and 11.4 Newton have been reported depending on the type of tissue or training task.^8,43,45-47^ These results underline the importance of quantitative objective force-based assessment of tool-tissue interaction and tissue manipulation skills. Although the importance of the assessment of tissue trauma and tissue manipulation has been shown, the implementation of force-based assessment is limited to only a few reports in the total of 35 included studies. A potential explanation for this is that the development and validation of this kind of safety-related metrics is relatively new and it takes time and money before software and hardware systems required to measure and process force data can be integrated into existing devices and box trainers.

### Future Perspectives and Recommendations

To summarize, the use of data-driven assessment tools has become increasingly important as surgical education moves towards a competency-based approach. They have been shown to provide objective and quantifiable measures of technical skills in laparoscopic surgery. Several parameters, representing different domains of technical skill can be measured such as instrument movements and accuracy, safe tissue handling, and instrument-tissue interaction. Each tool has its strengths and limitations, and the choice of assessment tool should depend on the specific learning objectives and context. It is advised to incorporate data-driven assessment and to utilize combined force- and motion-based assessment as objective measures of technical competency, to ensure patient safety and to improve surgical outcomes.

It’s important to note that the success of integrating data-driven assessment tools relies on several factors, including the validity and reliability of the assessment metrics, and the feedback and guidance provided to trainees based on the result of the assessments. Ackermann et al. investigated factors influencing surgical performance, and concluded that it is necessary to determine training measures (specific tests and selection procedures) that help to overcome the individual learning curve.^48^ The present result show that numerous measures exist.

This systematic review shows that the majority of literature described development and validation studies (Figure 1). Moreover, some of the included assessment tools had up to seven different cross-references to show one type of validation (Table 2). To prevent wasteful spending of funds and resources through excessive development and experimentation, without subsequent integration into skills training curricula, program directors and surgical trainers should collaborate closely to establish consistent regulations and standards governing the training and assessment of skills in minimally invasive surgery.

Assessment tools for surgery have significantly improved the quality of surgical care by providing an objective evaluation of skills and abilities, resulting in a reduction in errors and increased efficiency.^1,3,49,50^ Both trainees and experienced surgeons can utilize these tools to pinpoint areas for improvement and monitor progress. Pre-course, specific metric benchmarks or training goals can be set based on experience level. Peer comparison fosters competition and motivates autonomous training.^12,33^ Immediate feedback from these tools enables trainees and trainers to identify improvement areas and track progress efficiently. This aligns with deliberate practice principles, optimizing learning curves by targeting specific technical skills for competency.^34,51^ It’s recommended to integrate validated assessment tools early in surgical training and maintain their use throughout residency, fellowship, and advanced procedure training, supporting lifelong learning and development principles.

## Conclusions

This systematic review highlights the critical yet underutilized role of data-driven assessment tools in training for minimally invasive surgery. Despite the proven benefits of these tools for objective skill evaluation, their integration into training programs remains limited. To enhance surgical training, program directors and trainers should actively incorporate validated, metric-based tools into curricula, providing direct feedback and ensuring trainee proficiency before clinical practice. The combination of force- and motion-based assessment tools should be considered for a more comprehensive assessment of technical skills.
